# Effective multi-sectoral approach for rapid reduction in maternal and neonatal mortality: the exceptional case of Bangladesh

**DOI:** 10.1136/bmjgh-2022-011407

**Published:** 2024-05-06

**Authors:** Aniqa Tasnim Hossain, Elizabeth A. Hazel, Ahmed Ehsanur Rahman, Adam D. Koon, Heather Jue Wong, Abdoulaye Maïga, Nadia Akseer, Yvonne Tam, Neff Walker, Safia S. Jiwani, Melinda Kay Munos, Shams El Arifeen, Robert Black, Agbessi Amouzou

**Affiliations:** 1 International Centre for Diarrhoeal Disease Research, Bangladesh, (icddr,b), Dhaka, Bangladesh; 2 International Health Department, Johns Hopkins University Bloomberg School of Public Health, Baltimore, Maryland, USA

**Keywords:** Maternal health, Obstetrics, Health policy, Child health, Health systems evaluation

## Abstract

**Background:**

Bangladesh experienced impressive reductions in maternal and neonatal mortality over the past several decades with annual rates of decline surpassing 4% since 2000. We comprehensively assessed health system and non-health factors that drove Bangladesh’s success in mortality reduction.

**Methods:**

We operationalised a comprehensive conceptual framework and analysed available household surveys for trends and inequalities in mortality, intervention coverage and quality of care. These include 12 household surveys totalling over 1.3 million births in the 15 years preceding the surveys. Literature and desk reviews permitted a reconstruction of policy and programme development and financing since 1990. These were supplemented with key informant interviews to understand implementation decisions and strategies.

**Results:**

Bangladesh prioritised early population policies to manage its rapidly growing population through community-based family planning programmes initiated in mid-1970s. These were followed in the 1990s and 2000s by priority to increase access to health facilities leading to rapid increases in facility delivery, intervention coverage and access to emergency obstetric care, with large contribution from private facilities. A decentralised health system organisation, from communities to the central level, openness to private for-profit sector growth, and efficient financing allocation to maternal and newborn health enabled rapid progress. Other critical levers included poverty reduction, women empowerment, rural development, and culture of data generation and use. However, recent empirical data suggest a slowing down of mortality reductions.

**Conclusion:**

Bangladesh demonstrated effective multi-sectoral approach and persistent programming, testing and implementation to achieve rapid gains in maternal and neonatal mortality reduction. The slowing down of recent mortality trends suggests that the country will need to revise its strategies to achieve the Sustainable Development Goals. As fertility reached replacement level, further gains in maternal and neonatal mortality will require prioritising universal access to quality facility delivery, and addressing inequalities, including reaching the rural poor.

**WHAT IS ALREADY KNOWN ON THIS TOPIC**

- Maternal and child mortality has decreased dramatically around the world in the previous twenty years; however, progress is uneven and has stalled in some regions.

**WHAT THIS STUDY ADDS**

- Through this in-depth case study, we found that interventions delivered at the time of delivery, along with decreasing fertility risks facilitated mortality reductions.
- Bangladesh’s multi-sectorial and persistent focus on maternal and newborn health, along with economic and infrastructural improvements allowed for rapid gains.

**HOW THIS STUDY MIGHT AFFECT RESEARCH, PRACTICE OR POLICY**

- Understanding the national policy and programming drivers of mortality reduction will inform efforts for further and sustained improvements in health.

## Introduction

 Over the past three decades, Bangladesh has achieved impressive progress in health and population indicators, including one of the fastest declines in maternal and neonatal mortality among low- and middle-income countries (LMICs). The United Nations (UN) estimates suggest a steady decline in neonatal mortality rate between 1975 and 1990, with an average annual rate of reduction (AARR) of 2.4%. The pace doubled after 1990 with an AARR of 4.0% during 2000–2010 and 5.2% for 2010–2020.^1^ The reduction in maternal mortality was 5.2% from 2000 to 2010 and then 5.7% over the subsequent decade.^2^ These declines are remarkable in light of the fact that Bangladesh qualified as a low-income country through much of this period, all the way up until 2015 when it reached middle-low income status.^3^

Located in South Asia, Bangladesh is one of the most densely populated countries in the world with 163 million people—1240 people per km^2^—in 2019.^4^ This rapid growth and high population density led the country to take early measures to adopt and scale-up family-planning programmes. As of 1990, women had, on average, 4.5 children over their lifetime and this dropped to 2.0 by 2019, reducing population growth from 2.4% per year to 1%.^5^ Nonetheless, the population doubled between 1980 and 2019 due to population momentum and reduced mortality. Life expectancy has risen by a striking 16.7 years between 1990 and 2020.^5^ In the past three decades, Bangladesh has had extraordinary economic growth and social development. Gross domestic product (GDP) per capita rose from $306 in 1990 to $418 in 2000 and $1856 in 2019. The poverty rate fell by more than a third, with the proportion of the population living under the poverty line ($1.90/day) dropping from 44% in 1990 to 14% in 2016.^3^

Bangladesh stands out as an exemplar country in the South Asia region for reduction in neonatal mortality rate, with the fastest decline—4.6% reduction per year, from 44 deaths per 1000 live births in 2000 to 17 in 2020 (online supplemental figure 1). The maternal mortality reduction in Bangladesh is comparable to India and Nepal in the region: 434 maternal deaths per 100 000 live births in 2000 to 173 in 2017 (5.4% annual reduction). How did Bangladesh achieve this progress in spite of its relative poverty? What were the driving factors behind its success? The answers to these questions will not only help Bangladesh maintain its impressive trajectory; they will also furnish evidence and lessons for similarly situated countries looking to emulate its gains.

We examined this through an in-depth case analysis; a mixed-method assessment of the drivers of maternal and neonatal health achievements in the previous two decades. We used a comprehensive framework that seeks to identify distal and intermediate levers and proximate factors that drove the rapid decline in maternal and neonatal mortality.^6^ Using this framework, we mapped available programmes, policies, health and non-health data to these levers/factors and examined the data where available. We systematically evaluated trends in maternal and neonatal mortality since 2000 and assessed the main causes of deaths. We analysed relevant health system and policy inputs, changes in macro-level and household-level context, and intervention coverage and quality of care across the continuum of care with equity disaggregation by socioeconomic status.

## Methods

### Data sources

We analysed twelve household survey datasets (eight Demographic and Health Surveys (DHSs), one Multiple Indicator Cluster Survey (MICS) and three Maternal Mortality Surveys (MMS)), comprising 1.3 million births in the 15 years preceding the survey, to generate empirical estimates of neonatal mortality rates (NMR) at national and subnational levels using the full birth history module and covering the period 1993–2017.^7^,^15^ Coverage and equity of maternal and newborn health (MNH) services, and nutritional status and practices were also calculated from these household surveys. We used estimates of tetanus protection at birth produced by WHO/UNICEF.^16^ Facility readiness to provide quality antenatal care (ANC) and delivery services was computed using health facility assessments in 2014 and 2017.^17 18^

We searched the PubMed and Scopus databases and conducted a desk review of documents pertaining to health system organisation, human resources for health, and primary programme and policy documents—both with a focus on MNH and nutrition in Bangladesh since 1990. Finally, we conducted 19 in-depth key informant interviews and two focus group discussions on health system context and organisation, policy and programme development, and successes and challenges from the past 20 years of MNH in Bangladesh. Key informants and focus group participants included experts from government, non-governmental organizations (NGOs) and professional associations selected for their expertise on MNH, through informal, professional networks of the authors.

### Analyses

To estimate the NMR, we pooled all survey data with each dataset restricted to births in the past 15 years to increase the sample size and produce estimates for 3 years reference periods. Data were pooled after assessing the level of consistency in the mortality trends across surveys. The pooled data included a total of 1 245 501 births. We calculated standard error and 95% confidence interval of the mortality estimates using the Jackknife non-parametric method.^19^ We analysed inequalities in neonatal mortality by socioeconomic and demographic factors and described trends according to place of delivery, type of delivery (vaginal or caesarean section (c-section)), and birth risks based on women’s age, parity and birth intervals (see online supplemental table 1 for the definition of birth risk categories). Estimates of maternal mortality ratio (MMR) and causes of maternal death were obtained from the reports of the three MMSs. Causes of death among neonates came from recent estimates from the WHO’s Maternal Child Epidemiology Estimation Group.^20^

Coverage and equity indicators of maternal and newborn health, nutritional status and breast feeding practices were calculated with consistent definitions over time by the International Center for Equity in Health.^21^ An equity stratified analysis was done by household asset-based wealth quintile, urban and rural locality, maternal education, maternal age, geographic region. ANC facility readiness was computed as an arithmetic average of the availability of 18 essential items across five domains for ANC: equipment, diagnostics, medicines and commodities, basic amenities and human resources following the methodology described by Sheffel and colleagues.^22^ The core facility readiness score for delivery services was calculated from 13 items across four domains: equipment, medicines and commodities, guidelines, and human resources. In addition, four infection control and two basic amenities items were added to create a more expanded measure of delivery readiness which takes into account the availability of key infection control items.

Interviews and focus group discussions were transcribed and coded using NVivo software, in order to organise the analysis by main health system and emerging themes and along drivers' categories of the conceptual framework.

Three separate analyses were carried out to quantify the contribution of distal, intermediate and proximate factors to the decline in maternal and neonatal mortality. We first quantified the contribution of decline in fertility to maternal and newborn lives saved between 2000 and 2017, and to the observed decline in MMR and NMR during the same period using a method proposed by Jain.^23^ We then used the Lives Saved Tool (LiST) to estimate the impact of health intervention coverage change on maternal and newborn lives saved.^24^ LiST is a multi-cause mortality modeling tool for maternal, neonatal, child mortality and and stillbirth rates. It models the impact of scaling up coverage of proven health interventions to reduce cause-specific mortality directly, or via reducing risk factors for cause-specific mortality. Finally, we implemented a regression decomposition of the effect of distal, intermediate and proximate factors on changes in neonatal mortality (online supplemental annex 1).^25 26^ This decomposition allows for an analysis of multiple possible drivers of the change observed in an outcome of interest over time.^27^

### Patient and public involvement

It was not possible to involve patients or the public in the design, conduct, reporting or dissemination plans of this research. The findings of the study were shared with the Ministry of Health and stakeholders.

## Results

### Mortality

**Figure 1:**
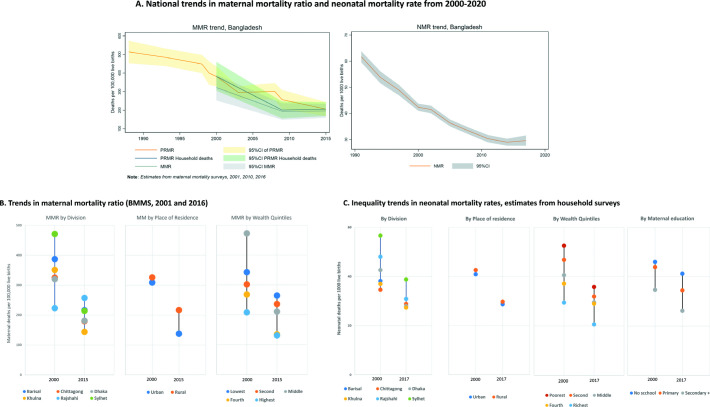
National trends and inequities in maternal and newborn mortality from Bangladesh Maternal Mortality Surveys (BMMS), 2000−2020. NMR, neonatal mortality rates; MMR, maternal mortality ratio; PRMR, Pregnancy related mortality ratio.

Contrary to the modelled UN estimates, the survey-based estimates suggest a slowing trend or even a stall in the pace of both maternal and neonatal mortality since around 2010. MMR declined from 322 deaths per 100 000 live births in 2000 to 194 in 2009 and 196 in 2015 while neonatal mortality declined from 42.4 deaths per 1000 live births in 2000 to 30.3 in 2011 and 29.6 in 2017 according to household survey data (figure 1).

Inequalities in MMR have reduced across divisions and wealth quintile but the gaps between urban and rural increased due to faster decline in urban areas (figure 1). MMR declined in all divisions, except in Rajshahi where it rose slightly. Sylhet had the highest MMR in 2000—but experienced the fastest reduction by 2015, almost closing the gap with other divisions (figure 1). NMR declined in all groups, however, the inequality gap reduced most substantially only by administrative division, closing the gap by 2017 between all divisions, except Sylhet where it remained higher despite the fastest decline.

The analysis indicates no NMR gap by urban or rural place of residence in 2000, a pattern that was maintained throughout until 2017. NMR gap by wealth quintiles closed little between 2000 and 2017. However, the inequality patterns changed, going from a linear pattern to a ‘top inequality’, with the richest quintile making more progress than the remaining 80% of the population. NMR inequality by education appears to have increased, with the fastest decline among better-educated women (figure 1).

**Figure 2:**
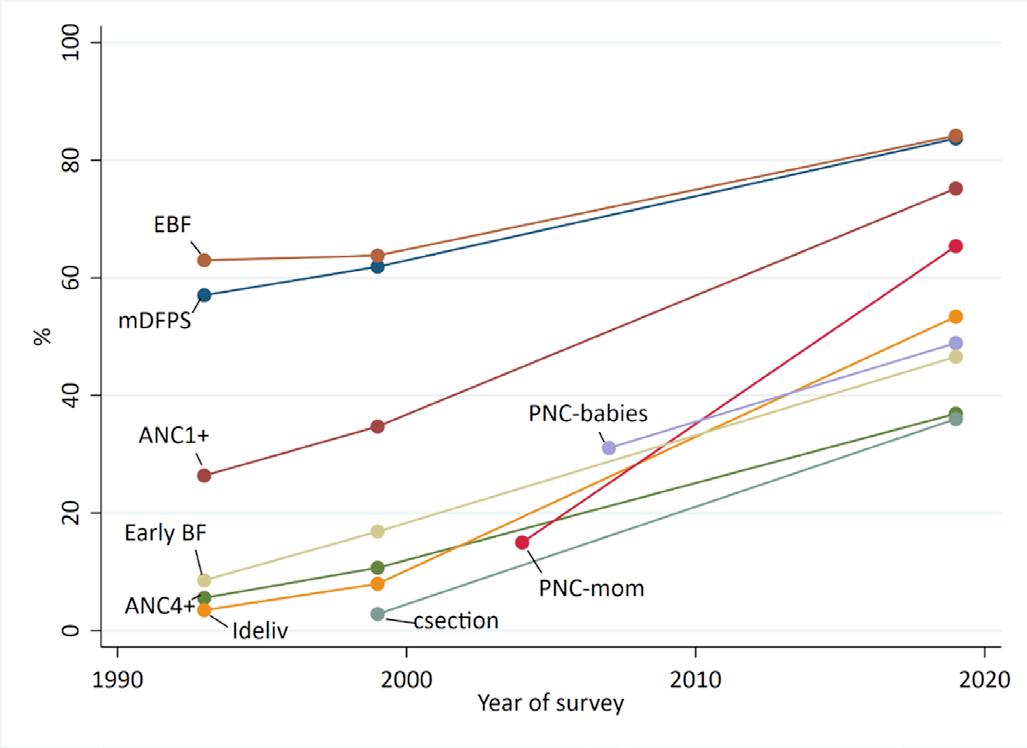
Trends in reproductive and maternal health intervention coverage indicators, 1993, 1999 and 2019. ANC1+, one or more visits of antenatal care with a skilled provider; ANC4+, four or more visits of antenatal care; c-section, cesarean sections; Early BF, baby was breastfed in the first hour after delivery; EBF, infants less than 1 month of age received only breastmilk in the previous 24 hours; ideliv, birth occurred at a health institution/health facility; mDFPs, demand for family planning satisfied with modern contraceptive methods; PNC-babies, baby received a postnatal check-up within 2 days postdelivery; PNC-mom, women received a postnatal check-up within 2 days postdelivery; TT, baby was born protected from tetanus toxoid infection.

There was no statistically significant difference in the risk of neonatal mortality between facility births and home births nor between vaginal and c-section delivery,except in 2015 where NMR for vaginal deliveries was higher than that of c-section deliveries (online supplemental figure 2 and 3). Births with minimal risk (ie, births of order two or three to women 18–34 years of age and birth interval >23 months) had the lowest mortality rate while those with multiple risks had the highest, although the mortality rate appeared to have stagnated among the former since 2005 (online supplemental figure 4).

### Causes of death

Based on verbal autopsy data, direct obstetric causes (primarily haemorrhage, eclampsia, obstructed labour and abortion), were responsible for two-thirds of maternal deaths in 2016 and showed limited improvements since 2010 where they caused 59% of deaths. Twenty per cent of maternal deaths were attributed to indirect causes in 2016, a substantially lower proportion compared with 2010 (35%) (online supplemental figure 5). An increased proportion and rate of deaths attributed to abortion-related complications (which counted for only 1.0% of maternal deaths in 2010, but 7.0% in 2016) was noticeable. Cause-specific estimates of neonatal deaths in 2000 and 2017 indicate major declines in prematurity; birth asphyxia and birth trauma; sepsis and other non-respiratory infections; and acute respiratory infections—with especially steep declines among the top three (online supplemental figure 6).

### Coverage of health interventions: contact, content, quality and equity

National coverage of MNH interventions was very low in 2000 but increased substantially by 2019. At least one ANC contact (ANC1+) with a qualified provider increased more than twofold from 35% in 1999 to 75% in 2019, and four or more ANC contacts rose more than threefold from 11% to 37% by 2019 (figure 2). More than half of women who got any ANC made at least one visit at a private for-profit sector facility (67% in 2017, increasing from 51% in 2014). Women were also more likely to have received basic interventions during ANC. Among all women who received any ANC, more reported blood (39%–74%) or urine (47%–77%) samples taken in 2019 compared with 1999 (online supplemental figure 7).

Even with the increase in ANC coverage, socioeconomic inequity gaps have been maintained over time. ANC1+ coverage increased from 21.1% to 49.6% for the poorest women and 70.6% to 95.1% for the wealthiest women, but the absolute coverage gap stayed almost the same (49.5 percentage points (pp) in 1999 vs 45.5pp in 2019) (online supplemental figure 8a). However, the urban-rural ANC1+ absolute gap decreased from 30pp in 1999 to 15pp in 2019 (online supplemental figure 8b). Readiness-adjusted coverage for ANC4+ increased from 17.7% in 2011 to 35.1% in 2017, however, this was attributable solely to the increase in crude coverage as the ANC readiness score did not change between 2014 and 2017.

In 1999, less than 10% of women delivered in a health facility, increasing to 53% by 2019 (figure 2). Among facility deliveries, more women delivered in the private sector (3% in 1999 to 38% in 2019), compared with the public sector (5% in 1999 to 16% in 2019 (online supplemental figure 9). Public hospitals had the highest facility-readiness scores for labour and delivery (72% readiness score in 2017), with moderate levels of readiness at private facilities (59% in 2017), and little change observed between 2014 and 2017 (online supplemental figure 10). C-sections increased from 3% to 34% in 2019 (figure 2). In 2019, 81% of private sector deliveries were c-section, compared with 35% of public sector deliveries.

The inequity gap in coverage of facility births has increased, the patterns have moved gradually from largely top inequity to a linear pattern, consistent with an increase from a very low level of coverage. In 1999, there was no coverage gap by wealth, but by 2019 the coverage gap increased to 30pp (online supplemental figure 8a). Inequities by urban-rural residence improved slightly (online supplemental figure 8b). Facility deliveries in rural areas increased from 5% to 49%, and urban facility deliveries increased from 25% to 68%, reducing the urban-rural gap from 21pp to 18pp. In rural areas, there was also a dramatic increase in c-section deliveries, rising from 1.6% to 32.8% on the same period (online supplemental figure 8b). The c-section rate among rural-poor increased from 0.4% to 13.4% in 2017, while the rural-wealthiest increased from 6.7% to 60.7% in 2017.

### Quantifying the effects of distal, intermediate and proximate drivers of maternal and neonatal mortality decline: fertility decomposition, LiST and regression decomposition analysis

Fertility decline has a direct impact on maternal and newborn lives saved through both the decline in birth rates and decline in the number of high-risk births. The fertility decomposition analysis showed that reduction in high-risk births contributed to 39% of the 60% reduction in the MMR between 2000 and 2017 and 37% of the 56% decline in neonatal mortality rate between 2000 and 2019 (online supplemental figure 11). This reduction was caused by an increase in age at first birth and a decrease in short birth intervals, and high parity births (online supplemental figure 12).

The fertility decomposition analysis also showed that fertility impact—through decline in birth rates and high-risk births—contributed to saving 52% of total maternal lives saved in 2017, with 21% due to decline in birth rates and 31% due to reduction in high-birth risks (online supplemental figure 13). Similarly, 57% of newborn lives were saved in 2019 due to fertility decline, with 25% due to decline in birth rates and 32% due to reduction in high-birth risks (online supplemental figure 13).

**Figure 3:**
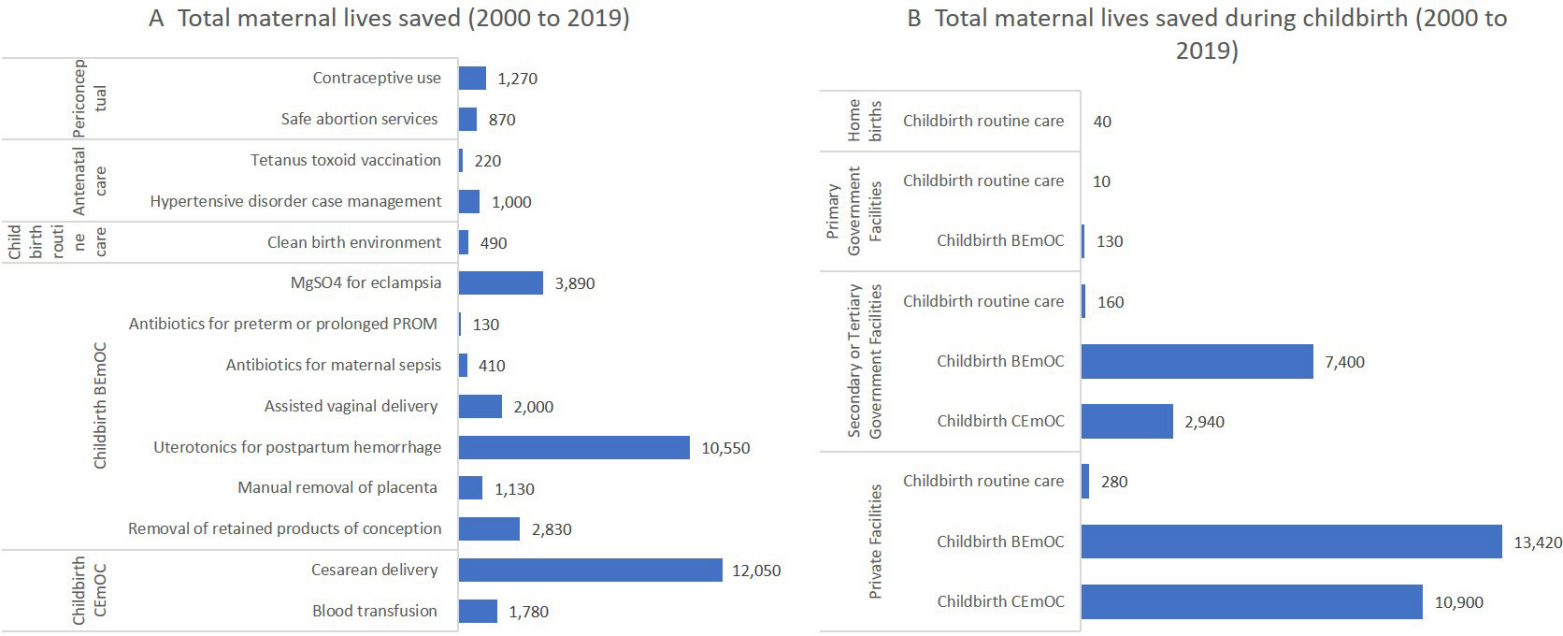
Maternal lives saved by interventions (**A**), groups of interventions by continuum of care and by facility types (**B**). BEmOC, basic emergency obstetric care; CEmOC, comprehensive emergency obstetric care; PROM, premature rupture of membranes.

We used LiST to attribute maternal and neonatal lives saved to health interventions. A total of 38 624 maternal lives were saved between 2000 and 2019 of which 35 265 (91%) were from interventions during childbirth (figure 3). Most maternal lives saved were due to basic emergency obstetric care (BEmOC) specific interventions during childbirth in government hospitals or private facilities. The top individual interventions contributors were c-section delivery, followed by uterotonics for post partum haemorrhage. The default assumptions used in LiST modelling only attribute lives saved to changes in facility delivery coverage. However, even if we make assumptions for the effective coverage of routine care interventions during childbirth at home, most maternal lives saved during childbirth were at private facilities, followed by secondary or tertiary government facilities. The LiST analysis estimated that changes in coverage of interventions contributed to 33% of the decline in MMR estimated by the United Nations Maternal Mortality Estimation Inter-agency Group (UN-MMEIG) between 2000 and 2017. LiST analysis estimated that MMR declined from 434 to 343 deaths per 10 000 live births, while UN-MMEIG estimated a decline from 434 to 173 in the same period (online supplemental figure 14).

**Figure 4:**
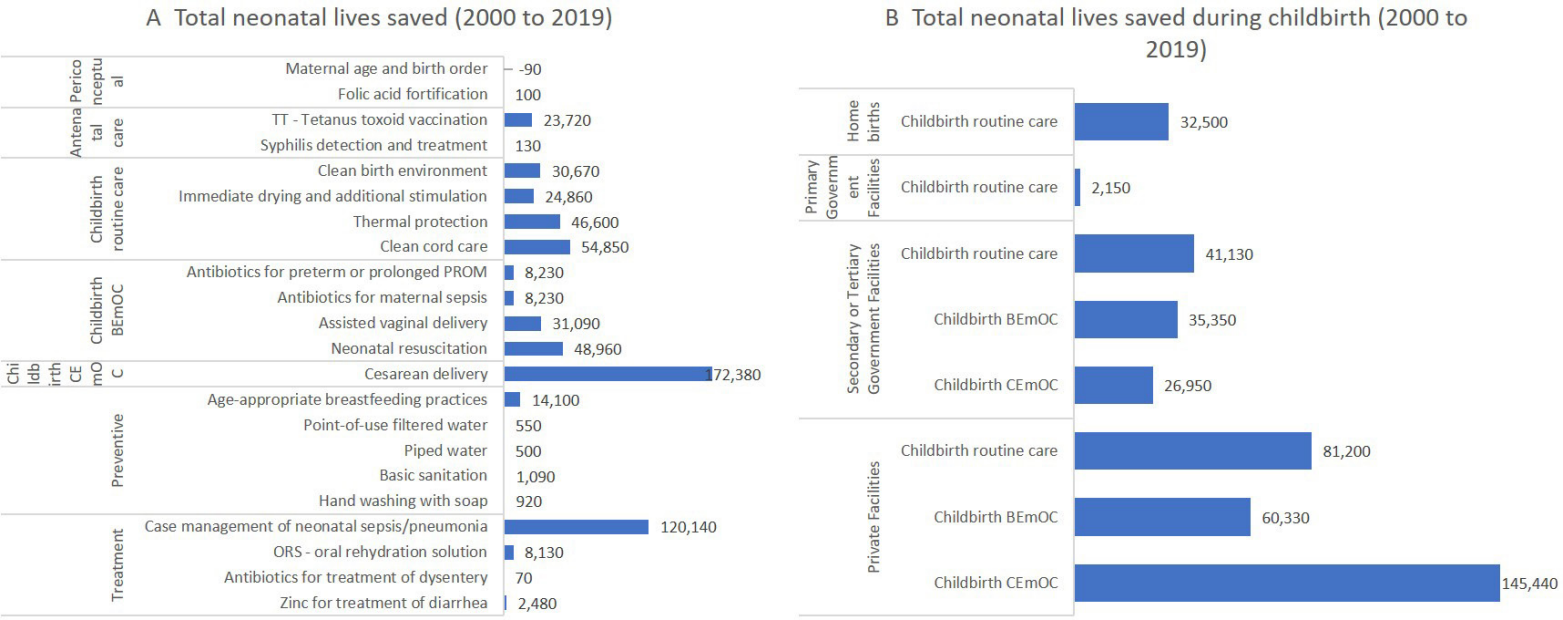
Neonatal lives saved by interventions (**A**), groups of interventions by continuum of care and by facility types (**B**). BEmOC, basic emergency obstetric care; CEmOC, comprehensive emergency obstetric care; PROM, premature rupture of membranes.

A total of 597 877 neonatal lives were saved between 2000 and 2019 of which 425 040 (71%) were due to interventions during childbirth (figure 4). Most neonatal lives saved were due to routine or BEmOC specific interventions during childbirth in government hospitals and routine and comprehensive emergency obstetric care specific interventions in private facilities. The top individual interventions that saved most neonatal lives were c-section delivery, followed by case management of sepsis or pneumonia. Most neonatal lives saved during childbirth were at private facilities, followed by secondary or tertiary government facilities. LiST estimated that changes in the coverage of newborn interventions contributed to 61% of the total neonatal mortality reduction estimated by the UN-IGME in the same period. NMR declined from 43 to 28 from 2000 to 2019, while United Nations Inter-agency Group for Child Mortality Estimation (UN-IGME) estimated a decline from 43 to 19 (online supplemental figure 15).

The regression decomposition analysis was performed for neonatal mortality only. The findings showed changes in proximate determinants (ie, changes in intervention coverage) had the largest contribution to the rapid decline in neonatal mortality, achieving two-thirds of the observed reduction (online supplemental figure 16). However, distal factors and intermediate factors also played significant roles. Changes in distal level factors, measured through community level socioeconomic variables, such as access to electricity, were associated with 25% reduction in mortality. Intermediate factors contributed to a 29% reduction in neonatal mortality and included indicators related to material circumstances, behavioural norms and decision-making, and health status and need.

### Programme and policy drivers

**Figure 5:**
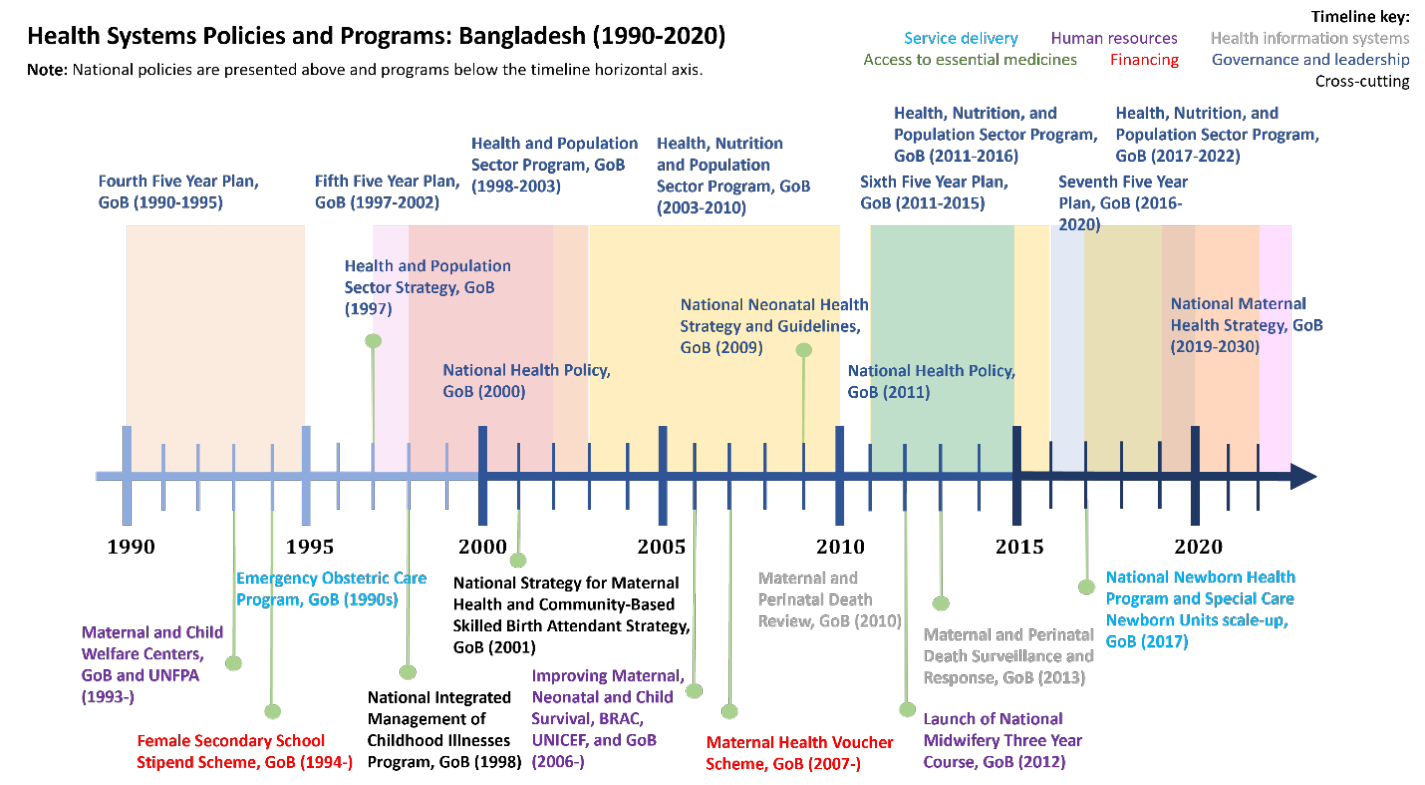
Maternal and newborn policy timeline. UNFPA, United Nations Population Fund; GoB, government of Bangladesh; BRAC, Building Resources Across Communities.

Much of the groundwork for successful MNH programming from 2000 to 2020 was laid through service delivery innovations in the decades that preceded our study time period (figure 5). The government emphasised the establishment of community clinics and led national scale-up efforts by facilitating public–private partnerships; recruiting and training providers; mobilising communities; and monitoring and quality assurance.^28^ These broad strategies and policies crafted for other health system functions represented innovations in MNH service delivery.^29^

However, human resources for health, including their supply, distribution and skill mix, continues to be a challenge in Bangladesh. While there has been a tremendous growth in private sector training and educational colleges (from zero private medical colleges in 1996 to 44 by 2011) and diagnostic centres (from 838 laboratories in 2013 to 5122 by 2012), these are disproportionately located in urban areas.^30^ In addition to privately licensed practitioners, there is a sizeable number of alternative care providers mostly distributed in rural areas.^31 32^

Total health expenditure in Bangladesh in 2015 was 2.9% of the GDP, one of the lowest allocations in the world.^33^ Revenue for healthcare is primarily derived through private sources, notably user fees and other out-of-pocket (OOP) payments, which constitute 93% of the nation’s private health expenditures.^34^

### Qualitative interviews

Respondents only vaguely touched on broader strategies and policies, preferring instead to describe the success of various programmes or interventions. There was widespread consensus that perhaps the strongest contributor to both maternal and newborn mortality reduction was the national family planning programme that has been in operation for several decades. Improved access to CEmONC, BEmONC, ANC and postnatal care were seen as important for mortality reduction.

Several interventions were viewed favourably, but because they were brought to scale in the latter part of our study time period, their effect on maternal and newborn mortality is uncertain. This includes the scale-up of chlorhexidine for cord care, the National Newborn Health Programme (NNHP) of which it is a part, Special Care Newborn Units, and the formalisation of midwifery as a distinct health cadre.

Views were mixed concerning several promising interventions. This included the Maternal Health Voucher Scheme (MHVS) because it is frequently mismanaged and restricted in coverage (by design it caters to the poorest districts). While the Helping Babies Breathe initiative, as part of the NNHP package has had some success, several interviewees felt it has yet to live up to its potential. The Maternal and Perinatal Death Surveillance and Response scale-up is polarising, with some feeling it has been a huge step forward and others thinking it has not contributed to health improvements. The rapid growth of c-section deliveries has also been acknowledged as a success, but because the growth exceeds need and is concentrated in private for-profits facilities, many worry that the relatively high costs negatively impacts equity. While some point to the expansion of misoprostol as a success, others suggest that it is too small scale of an intervention to make a meaningful impact on maternal mortality.

Some interventions or programmes were reported as missed opportunities. This includes the community-based skilled birth attendant scale-up, which never received the level of support it needed to establish trainees as legitimate birth attendants and were not seen as attractive options by the population. Interview respondents were somewhat perplexed by the lack of attention on any sort of programme to reduce stillbirths. To a lesser extent, many saw Kangaroo Mother Care as not living up to its potential, despite its promise (within the NNHP package). Finally, interviewees acknowledged that quality of care and facility readiness, particularly in the public sector, remain a problem.

### Improvement in non-health sectors: macro, household and individual levels

The population living in urban areas almost doubled since 1990, from 20% to 37%. Meanwhile, rural-development initiatives have led to almost universal access to electricity. Only 5% of rural Bangladeshis had electricity in 1993 increasing to 89% by 2019. Rural isolation was also lessened by advances in transportation and communications infrastructure. In the first decade of this century, Bangladesh built 19 000 km of dirt roads, 32 000 km of paved roads and 300 km of bridges.^35^ By 2017, 94% of households had a mobile phone, compared with only 32% as recently as in 2007 and 60% of women own their own phone.^36^

There has been significant improvement in the education, social standing and life prospects of Bangladeshi women. By 2019, female literacy was at 68%, an increase from 26% in 2000 and the proportion of women aged 15–49 attaining secondary or higher levels of education was 62%, increasing from 55% in 2007.^36^,^38^ Female labour-force participation increased from 25% in 1990 to 39% in 2019.

Women’s empowerment is both the cause and effect of deeper social changes within the household. The proportion of women 20–24 years of age who had been married by age 15 decreased from 38% in 2000 to 19% in 2017.^39^ By 2017, 75% of women said they were involved in decision-making about their own healthcare, a 26pp increase in less than two decades. Acceptance of domestic violence has decreased: 20% of ever-married women still said they believe a husband may beat his wife, a reduction from 36% in 2007.^36 38^

## Discussion

Bangladesh is an exceptional story of rapid decline in maternal and neonatal mortality in the past two decades. Our findings suggest an early adoption and persistent multi-sectoral approach that prioritised health as well as non-health socioeconomic and developmental factors. The programmatic drivers that we identified are consistent with those of an earlier case study of maternal mortality decline between 2000 and 2010.^40^

Four initiatives demonstrate how deliberate policy and programming designed to focus on MNH equity for marginalised communities can efficiently accelerate health gains.^41^ First, Bangladesh has benefitted from one of the world’s first, most consistent and best-organised sector-wide approaches (SWAPs), which replaced over 120 separate donor-financed programmes with 25 integrated, closely managed plans.^42^ Bangladesh’s SWAP has consistently focused on maternal/child health and family planning, enabling key advances such as the revitalisation of community clinics.^28^

Second, a pioneering piece of legislation in 1982 that prioritised essential generic drugs, positioned Bangladesh as the first low-income country to develop a pharmaceutical industry.^30^ This likely lowered the cost of care and spending for MNH, as now 97% of demand for medicines for all types of care are met by domestic manufacturing.^28^

A third innovation targeted specifically at increasing demand and access to reproductive healthcare for marginalised communities is the MHVS. This demand-side financing model grew from a pilot project, using Female Health and Family Welfare Assistants to identify pregnant women, issue a voucher and transport subsidy and provide reproductive health information in 500 of the poorest subdistricts.^43 44^

Finally, a long-standing education initiative, the Female Secondary Stipend and Assistance Programme, which provides conditional stipend and tuition assistance for girls in rural secondary schools, has likely strengthened MNH.^45^ In reaching over 2 million girls, the programme has cost-effectively helped to delay marriage, encouraged contraceptive use and reduced fertility.^45^ These initiatives demonstrate the power of investing intersectorally in social programmes that reduce health inequities.

Other non-health features like economic growth and development contributed to Bangladesh’s successes. Improvements in socioeconomic factors such as access to electricity, construction of a large network of dirt and paved roads have made it possible to open up rural areas and facility access to health facilities. Additionally, Bangladesh did not experience wide-scale and long-lasting humanitarian crises, disasters, conflict or war during these decades, allowing these investments in health and development to be fully realised.

The early and sustained investment in family planning programmes resulted successfully in fewer pregnancies and fewer high-risk pregnancies. Beginning in the 2000s there was a rapid escalation of facilities, including a network of community clinics that linked women to higher level facilities, and expansion of emergency obstetric care. By 2016, almost all women of reproductive age could reach a health facility within one hour.^14^ This expansion resulted in massive and rapid increases in facility usage for maternity and newborn care, including antenatal and delivery care. These increases were attributed largely to the major proliferation of private hospitals and clinics, which contributed to 70% of facility deliveries in 2019.

Our LiST analysis demonstrated the critical roles of interventions delivered during childbirth in saving most of the maternal and newborn lives during these two decades. Although almost half of births in Bangladesh occurred at home, most of the lives saved were attributed to births that occurred at facility levels, by interventions such as c-section delivery saving maternal and neonatal lives, and uterotonics and case management of neonatal sepsis or pneumonia saving maternal and neonatal lives, respectively.

An important question relates to the continued high level of home deliveries. We found that neonatal mortality reduced among both home and facility births. The massive increase in access to facilities in a context of high population density appeared to inhibit facility deliveries, in that, women would rather deliver at home, knowing that they could access a health facility quickly in case of a complication. Indeed, the 2016 MMS showed that 80% of women with pregnancy complications who did not seek care in a health facility reported that it was because the issues were not serious enough; only 2% reported constraints related physical access to facilities.^14^ Careseeking from health facilities for maternal complications increased from below 20% in 2001 to 46% in 2016. Consequently, mortality among home births decreased substantially, while mortality in facilities equally decreased in similar magnitude.

The proliferation of private hospitals and clinics and their excessive contribution to facility delivery has also caused a sharp increase in c-section, a third of all deliveries in 2019 were c-section. Across our quantitative models, this intervention was associated with the greatest decline in maternal and neonatal mortality. C-section may be considered as a proxy for higher levels of care, however, the rapid growth of c-section delivery by private providers was widely viewed as a source of concern because they increasingly exceed medical necessity and are expensive, placing unnecessary financial strain on households and potentially widening socioeconomic disparities.^46^

A critical question is whether the exceptional gains in maternal and neonatal mortality observed since 2000 are currently being sustained and improved on. According to the maternal and peri-neonatal transition model, Bangladesh progressed very fast from stage I (high maternal mortality, high fertility with predominantly direct causes of maternal deaths) to stage III (lower mortality and fertility with direct causes predominant) in less than two decades.^47^ To accelerate further, the country needs to quickly strengthen strategies that are consistent with transitioning to stage IV (low mortality and fertility, indirect causes are predominant). These include aiming for universal BEmONC and CEmONC, management of small and sick newborns, and focusing on left-behind population. This means that hospital deliveries must increase drastically, including in rural settings, and the priority for quality of maternity care encompasses antenatal, delivery and postnatal care. We found suboptimal ANC intervention coverage, indicating further prioritisation of quality and content of care is needed.^48^ At the same time, there is concern that unregulated growth of private for-profit facilities may lead to overuse of unnecessary interventions such as induction augmentation and c-sections. Moreover, high OOP expenses can put undue pressure on families and individuals, especially as health costs continue to escalate.^33 49^ Reducing inequalities in access to care, while regulating and strengthening existing service delivery models can accelerate gains.

Another striking contributing success factor in Bangladesh has been the persistent promotion of health data availability and use, including evidence-based programming based on piloting of interventions and strategies at small scale before national scale up.^50^ Many of the world’s leading strategies for addressing MNH in low-income settings have been developed and, in many cases, brought to scale in Bangladesh. Bangladesh is the only country among the LMICs to have implemented three massive household surveys for maternal health and mortality; and conducts at an exceptionally regular pace DHSs and MICSs.

Despite this, our study is limited in many parts by the unavailability of data. Bangladesh is rich in health data yet important gaps remain. We did not have baseline data for quality of care and service readiness, nor for many key neonatal care indicators. Most proven interventions during labour and delivery lack coverage measurements from household surveys. However, as the reduction of cause-specific mortality is linked to the effectiveness of interventions delivered during these contacts, for the LiST analysis, we estimated the coverage of these interventions from the use of services at facility, available from household surveys, and the proportion of facilities ready to deliver these interventions, available from facility surveys. We used c-section rates by wealth quintiles from household surveys, adjusted it to exclude elective c-section, and weighted it by total fertility rate and by wealth quintile. This approach helped correct c-section rate by excluding non-medical c-sections.

## Conclusion

Our research suggests there was no single policy, programme or intervention that resulted in these improvements but an accumulation of positive aspects and investments over time. Survey estimates show a slower decline in NMR and MMR in the past decade, calling for increased attention to MNH.

Accelerating mortality reductions will require improving substantially the coverage of ANC, skilled and facility delivery, postnatal care by improving equitable access and expanding service delivery points. Along with coverage, improving the readiness and quality of care at health facilities (BEmONC and CEmONC facilities) with critical attention to small and sick newborns, direct maternal causes (haemorrhage and eclampsia) and interventions around the time of birth. Threats to further gains, such as the high proportion of OOP health spending and rapid growth of private for-profit clinics underscore the importance of urgently expanding financial protection and regulatory oversight in order to sustain progress. In the next 10 years, Bangladesh must continue to reach the poor and most disadvantaged population with MNH interventions to close the coverage gap and pursue investment in social and economic programmes such as girls’ education, women empowerment, rural development and transportation.

## Supplementary material

10.1136/bmjgh-2022-011407 online supplemental file 1

10.1136/bmjgh-2022-011407 Uncited online supplemental file 1

## Data Availability

No datasets were generated for this study. All secondary data analysis was completing using data from a variety of publicly available datasets. All data are cited in this paper and are available from the third parties listed in the references. Qualitative data presented in this study may not be shared since the information cannot be adequately deidentified.
