# Supplementary material for: Effective multi-sectoral approach for rapid reduction in maternal and neonatal mortality: the exceptional case of Bangladesh
Source: BMJ Glob Health. 2024 May 6;9(Suppl 2):e011407. doi: 10.1136/bmjgh-2022-011407 (PMC11085986; doi:10.1136/bmjgh-2022-011407)
Supplement: online supplemental file 1 [file bmjgh-2022-011407supp001.pdf]

Annex:

Annex Figure 1. Estimates of maternal mortality ratio and neonatal mortality rates, United Nations Maternal Mortality Estimation Inter-agency Group (MMEIG), United Nations Inter-agency Group for Child Mortality Estimation (UN-IGME) and Institute for Health Metrics and Evaluation (IHME).

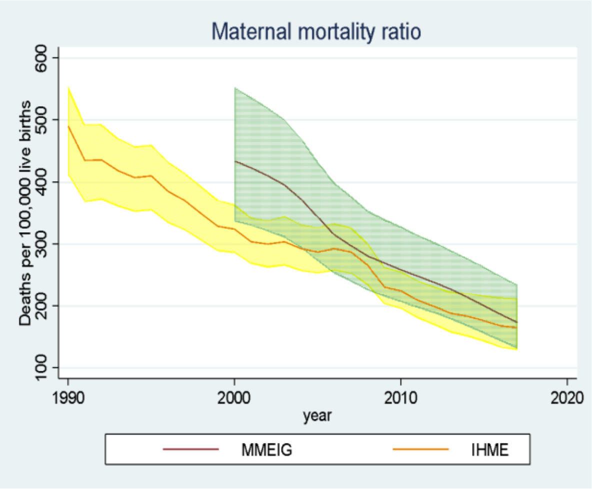

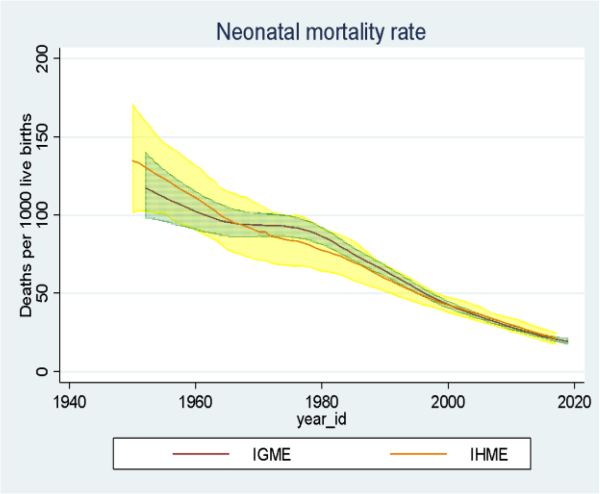

Category	Definition
Minimal risk	
No additional risk	Second and third birth order born to mother between age 18 and 34 years and birth interval > 23 months
Unavoidable risk	
First birth 18-34 years	First birth order born to mother between age 18 and 34 years
Single high-risk	
Mother's age < 18 years	Mother's age at birth < 18 years, birth order < 4 and birth interval > 23 months
Mother's age > 34 years	Mother's age at birth > 34 years, birth order < 4 and birth interval > 23 months
Birth interval < 24 months	Birth interval < 24 months, mother's age at birth into 18-34 years and birth order < 4
Birth order > 3	Birth order > 3, mother's age at birth into 18-34 years and birth interval > 23 months
Multiple high-risk	
Mother's age < 18 years and birth interval < 24 months	Mother's age at birth < 18 years and birth interval < 24 months or mother's age at birth < 18 years and birth order > 3
Mother's age > 34 years and birth interval < 24 months	Mother's age at birth > 34 years, birth interval < 24 months and birth order < 4
Mother's age > 34 years and birth order > 3	Mother's age at birth > 34 years, birth order > 3 and birth interval > 23 months
Birth interval < 24 months and birth order > 3	Birth interval < 24 months, birth order > 3 and mother's age at birth into 18-34 years
Mother's age at birth > 34 years, birth interval < 24 months and birth order > 3	Mother's age at birth > 34 years, birth interval < 24 months and birth order > 3

Annex Table A1: Definition of birth risk categories

Annex 1: Regression decomposition of drivers of neonatal mortality change in Bangladesh

Multivariable decomposition has been used to analyze a multitude of outcomes, including mortality. (1-9) Factors linked to mortality reduction in children (<5 years) include socioeconomic indicators,(1, 2, 5, 6, 8) birth spacing,(1, 4, 9) insecticide-treated bed nets,(3) reduced family size,(1) antenatal care,(1, 9) multiplicity of birth,(4, 9) and mother's age.(2, 9) Decomposition can allow for an analysis of multiple possible drivers of the change observed in an outcome of interest over time.(3) The typical application is as follows: to determine how much of the change in under-5 mortality is attributable to each factor, coefficients obtained from the multivariable analyses are multiplied by the changes in the determinants between the years studied. The products are added together, after which the sum is exponentiated. This provides the change in mortality as a result of changes in the determinants between surveys.(7, 9)

Approach

To conduct our analyses, we used two rounds of Bangladesh DHS from 1999-00 and 2017-18. These surveys are nationally representative and are based on a two-stage stratified household sample design.(10) We analyzed the dichotomous outcome of neonatal mortality, defined as death within the first 28 days of life using the conceptual framework.(11) At the distal level, we include community-level context variables including urban/rural residence, phone access, and access to electricity. At the intermediate level, we include variables related to material circumstances (maternal and paternal education, wealth index, annual income, religion, marital status, piped water, open defecation, improved water and sanitation, number of household members, people living per room, and sex of household head), behavioral norms and decision-making (diarrhea care-seeking, and acute respiratory illness (ARI) care-seeking), health status/need (risk categories related to maternal age, birth interval and birth order, delivery by c-section, maternal height and BMI, low birthweight of baby, history of giving birth to twins, sex and age of child, history of stillbirth, unwanted pregnancy, diarrhea, ARI, and fever prevalence), and program and service levers (facilities offering family planning, antenatal care, normal delivery, c-section, postnatal care, and facilities with electricity, water, toilet facilities, and essential drugs). At the proximal level, we consider variables related to intervention coverage, which include: four or more antenatal care visits (ANC4+), skilled birth attendance (SBA), health facility delivery (HFD), iron during pregnancy, drugs for intestinal parasites during pregnancy, blood pressure measured during pregnancy, urine sample taken during pregnancy, blood sample taken during pregnancy, use of a modern contraceptive method, and early introduction of breastfeeding.

We used the method outlined by Hong et al to conduct our decomposition of neonatal mortality in Bangladesh.(7, 9) Bivariate logistic regression was conducted to determine factors which were associated with neonatal mortality. Variables were included into the multivariable model if they had a p-value ≤0.25. We created a hierarchical multivariable model by including first distal-level factors through a process of backward elimination kept only those with a p-value≤0.15. We then introduced intermediate-level variables into the multivariable model and repeated the process of backward elimination. Finally, we conducted the same process with proximal-level variables. Coefficients in the multivariable model were then multiplied by the change in these variables between 2000-2018. These products were exponentiated, and this value was used to calculate the percent contribution of each factor using the following formula:

Percent contribution = (1-exponentiated product)/absolute value of percentage change in NMR

The percentage change in neonatal mortality rate (NMR) was calculated from within the survey datasets and also obtained from the UN Inter-agency Group for Child Mortality Estimation(12) for comparison. Complex survey design was accounted for by using Stata's *svyset* command. Models were adjusted for child sex and survey year. Analyses were completed using Stata SE 15.1.

Annex Figure 2: Trends in neonatal mortality rate by place of delivery

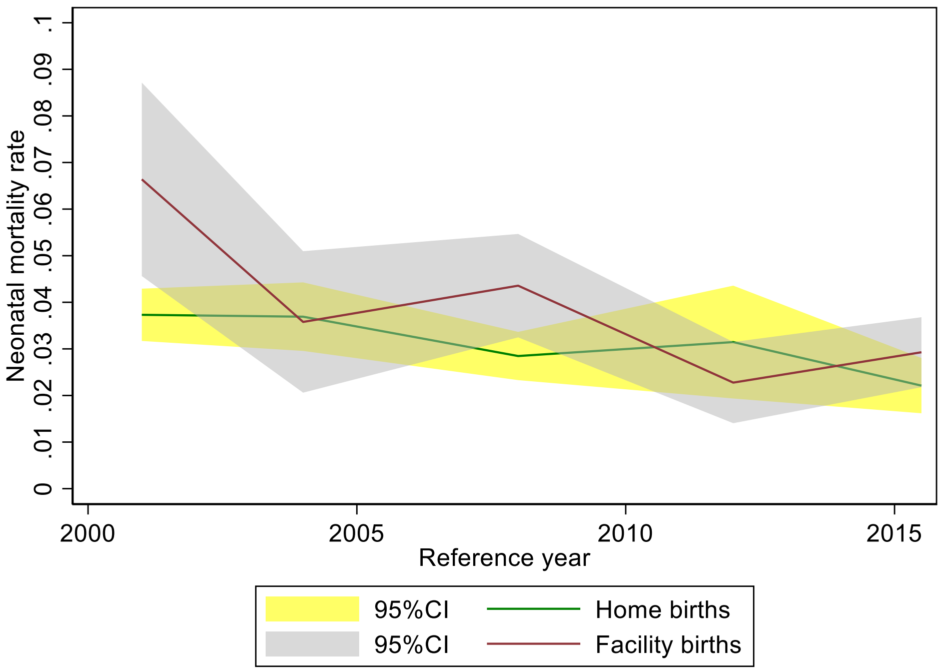

Annex Figure 3: Trends in neonatal mortality rate by type of delivery

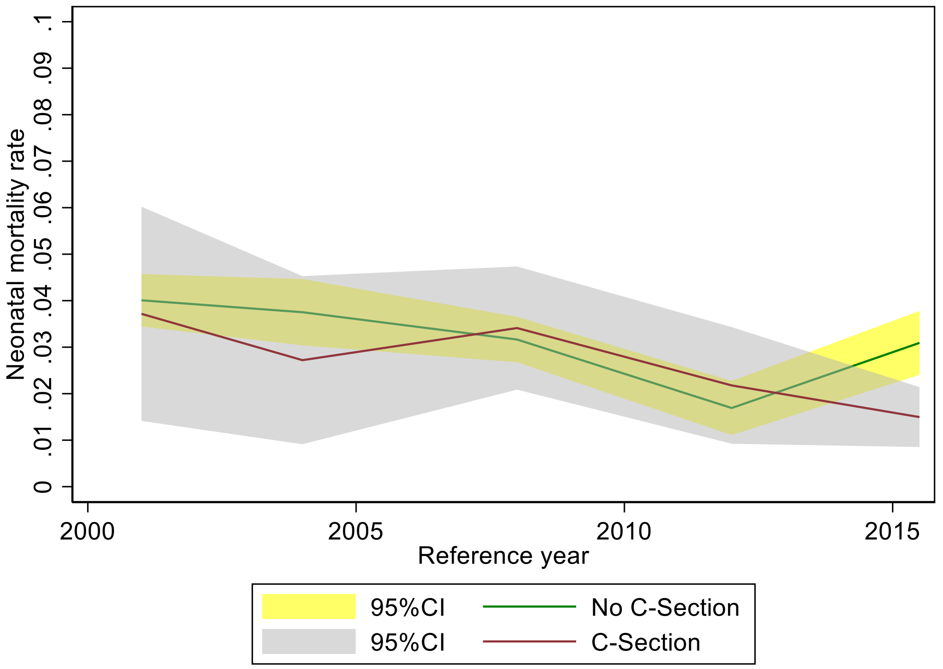

Annex Figure 4: Trends in neonatal mortality by birth risks

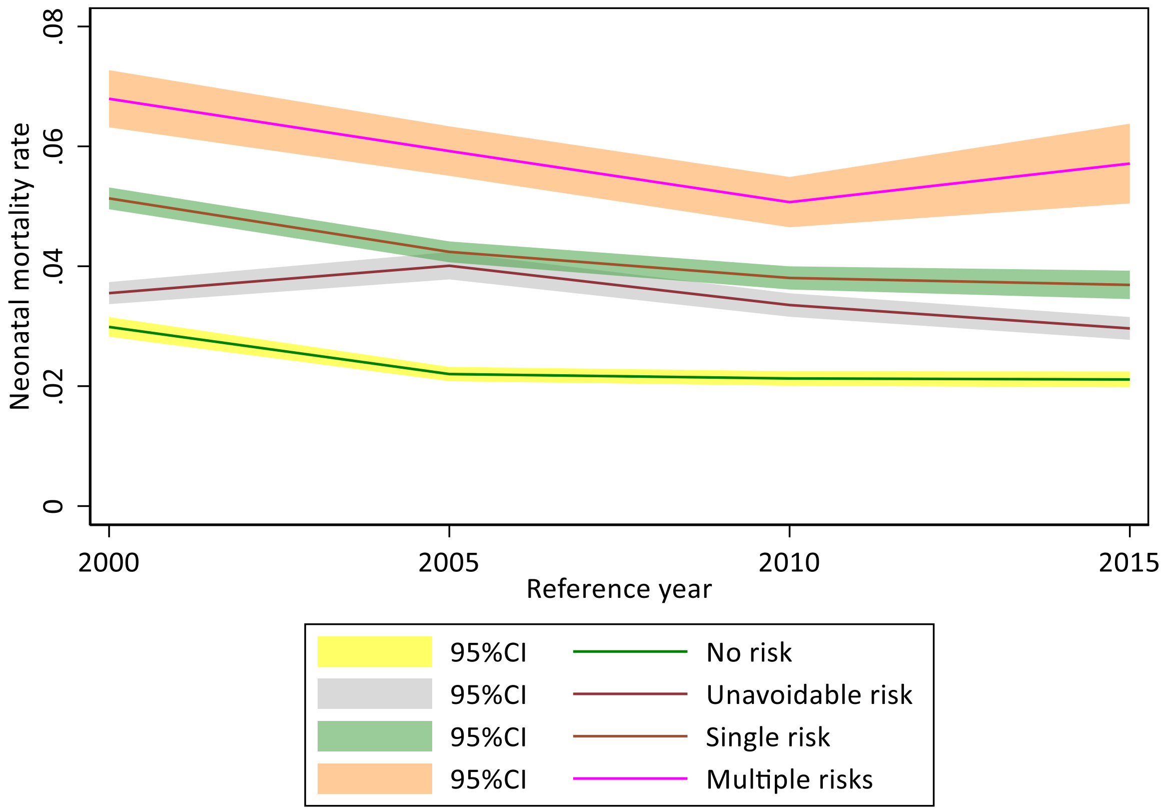

Annex Figure 5. Causes of maternal death in Bangladesh (Bangladesh Maternal Mortality Surveys (BMMS) 2010 and 2016)

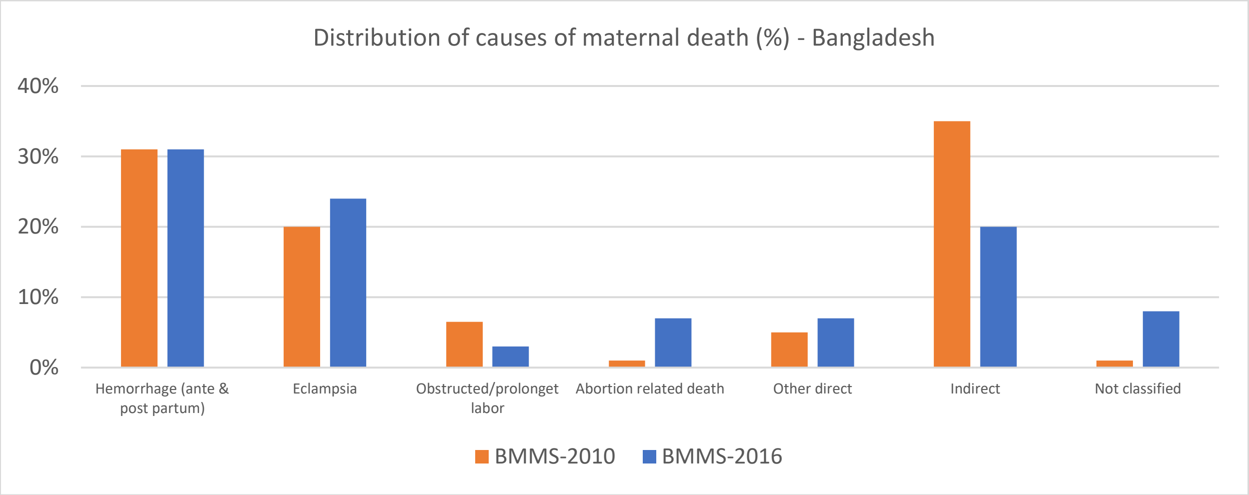

Annex Figure 6. Causes of neonatal death in Bangladesh, Maternal and Child Epidemiology Estimation Group (MCEE) estimates 2017

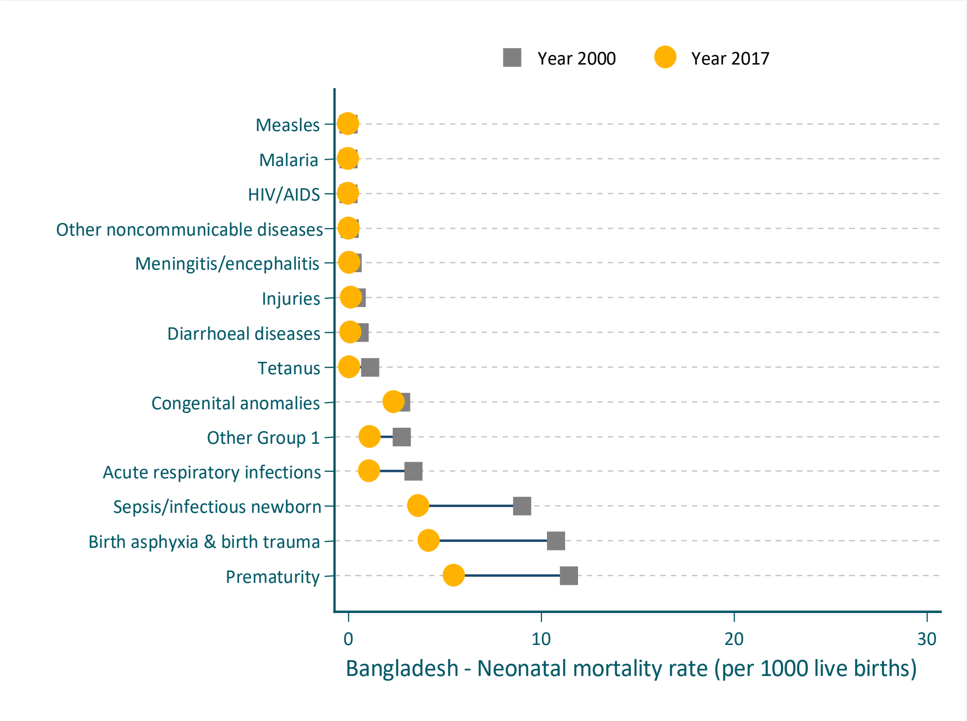

Annex Figure 7. Quality components of antenatal care, 1993, 1999 and 2019

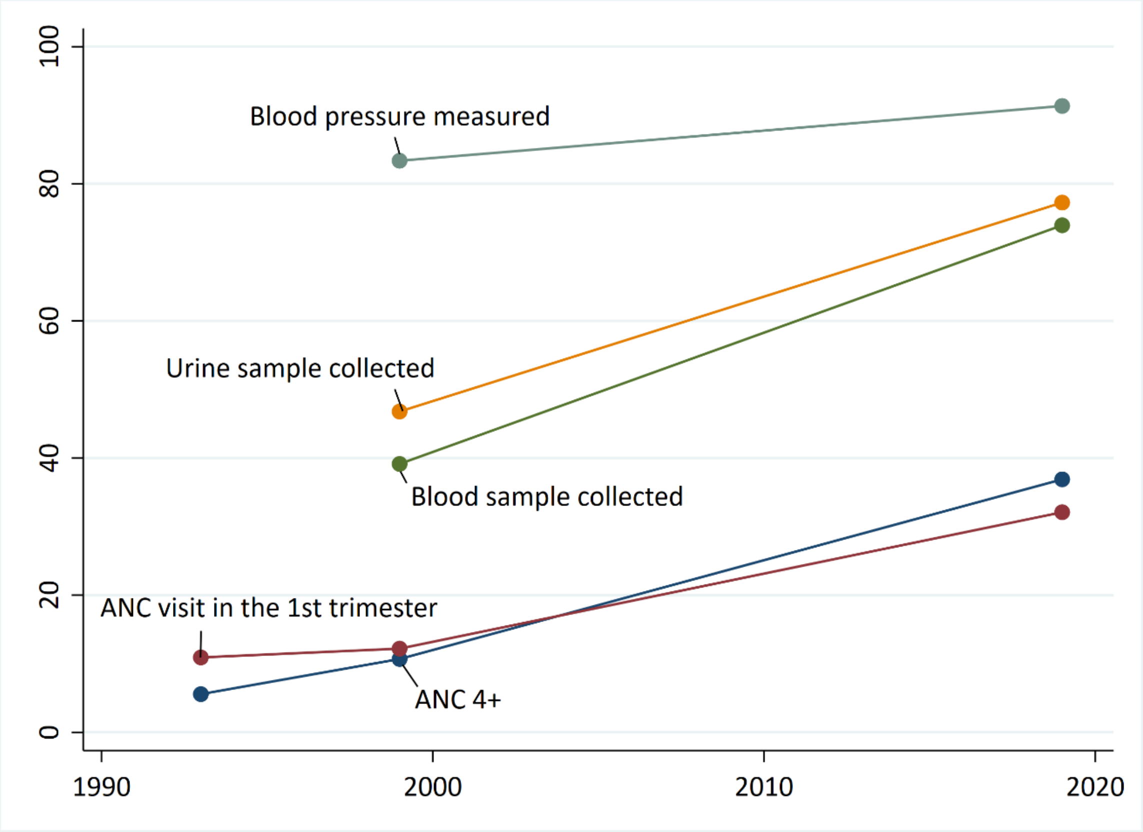

Annex Figure 8a. Coverage gap comparing the wealthiest women (Q5) and the poorest women (Q1) for selected family planning, maternal and newborn intervention and practice coverage, 1999, 2007, 2012, 2019.

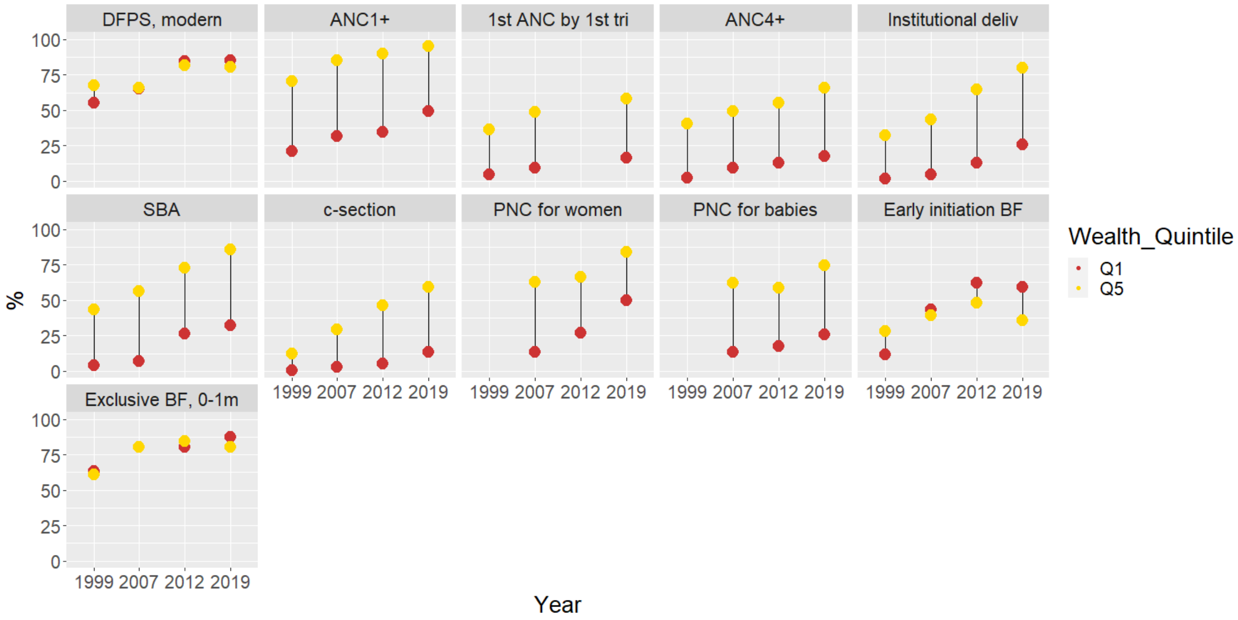

Annex Figure 8b. Coverage gap comparing the urban and rural residence for selected family planning, maternal and newborn intervention and practice coverage, 1999, 2007, 2012, 2019.

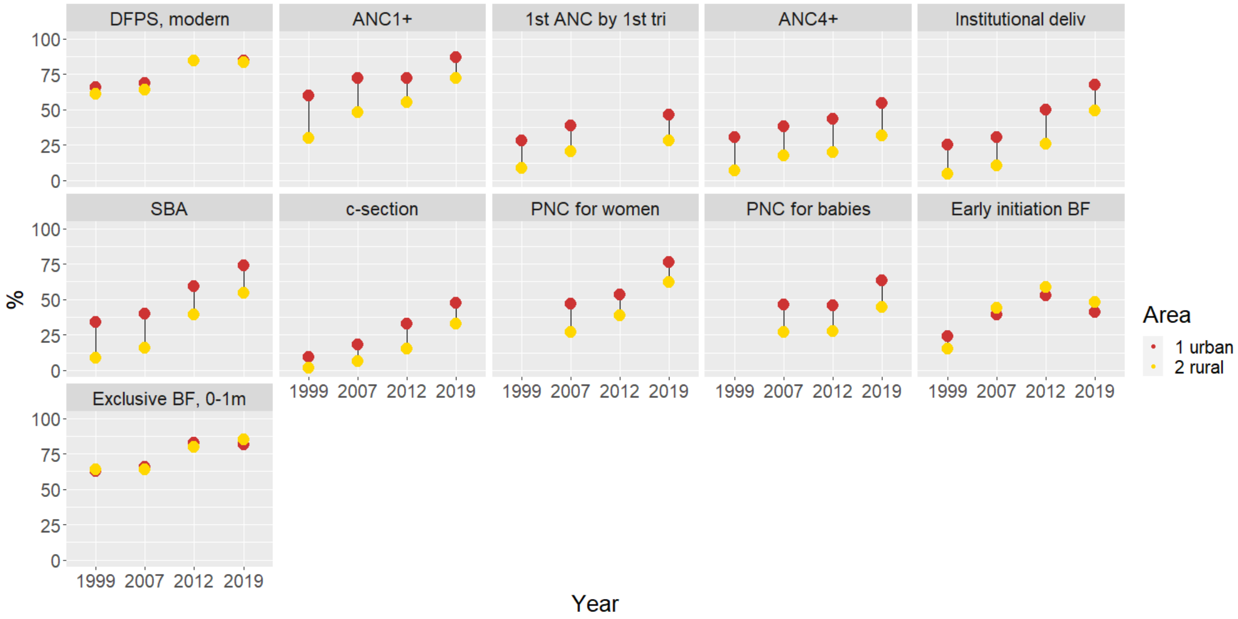

Annex Figure 9. Proportion of deliveries that occurred in public sector facilities, private sector facilities (for-profit and non-governmental organization (NGO), and outside the facility, 1999 – 2019.

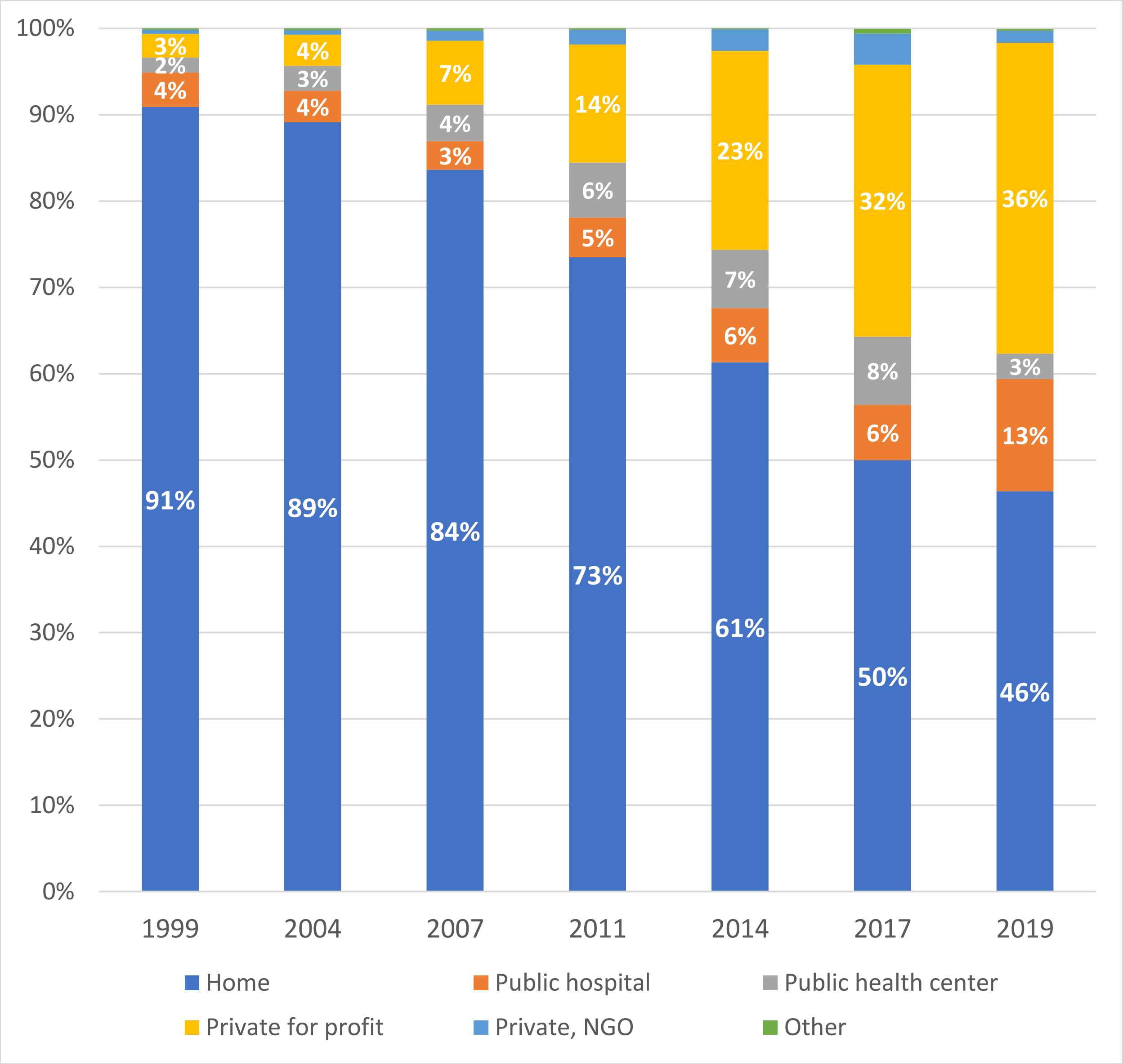

Annex Figure 10. Facility readiness for labor & delivery by facility type, 2014-2017.

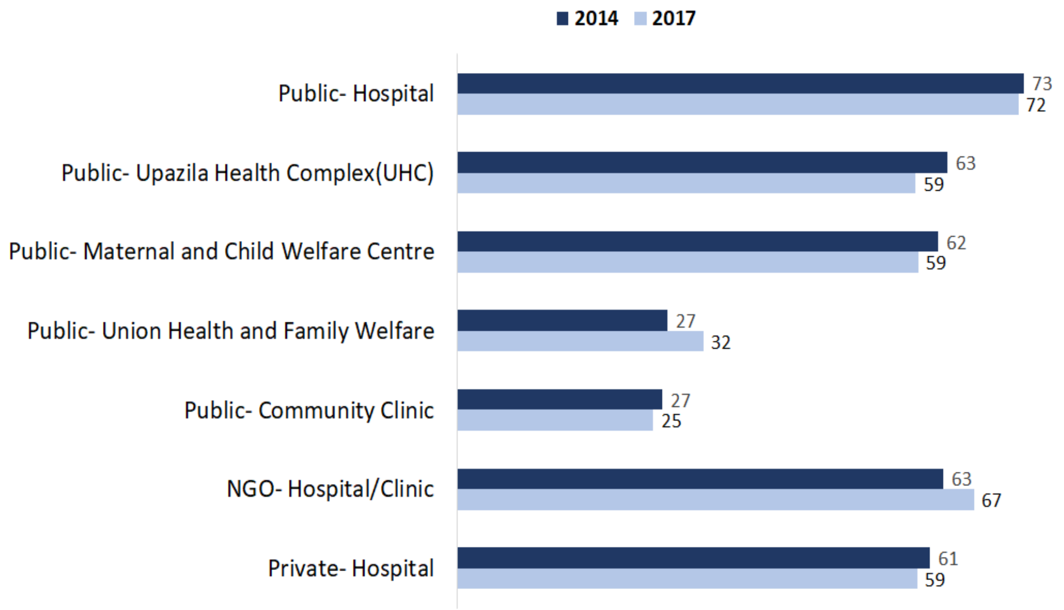

Annex Figure 11. Percent contribution of fertility decline to overall decline in maternal mortality ratio and neonatal mortality rate.

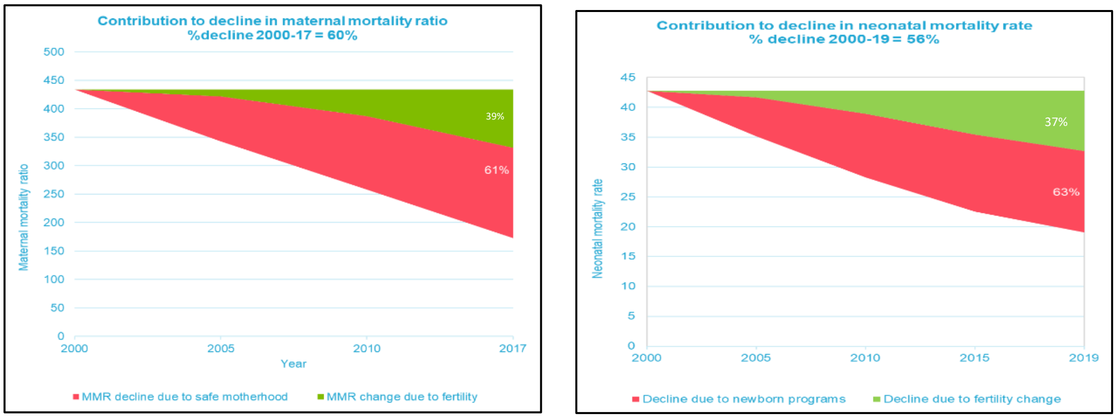

Annex Figure 12. Changes in the percentage of births by birth risk categories defined based on age at birth, birth interval, and birth order.

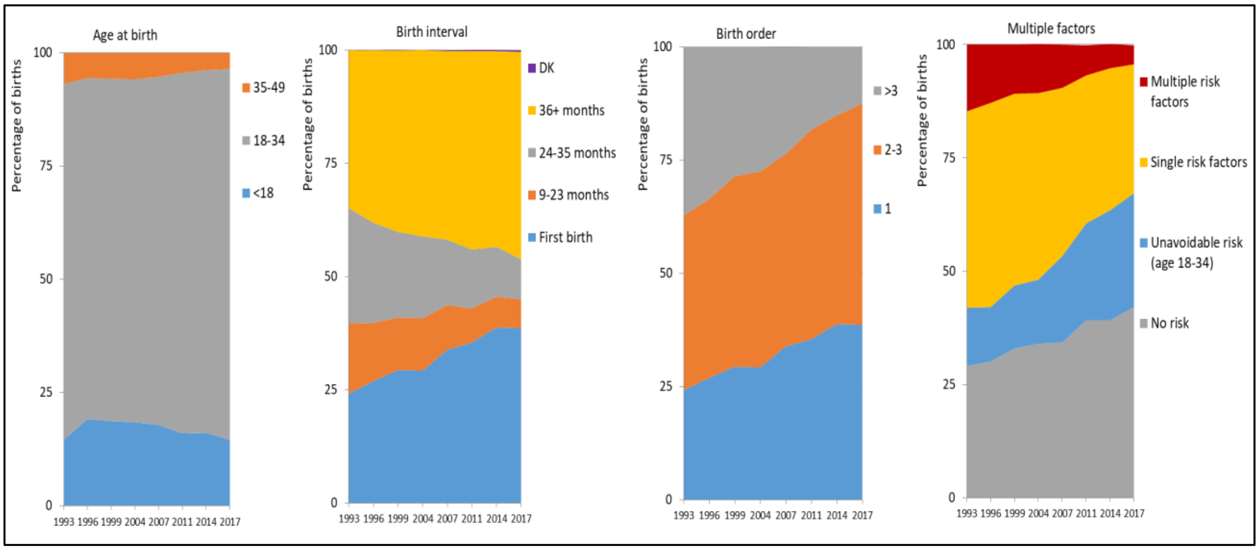

Annex Figure 13. Percent contribution of fertility decline to maternal and neonatal lives saved.

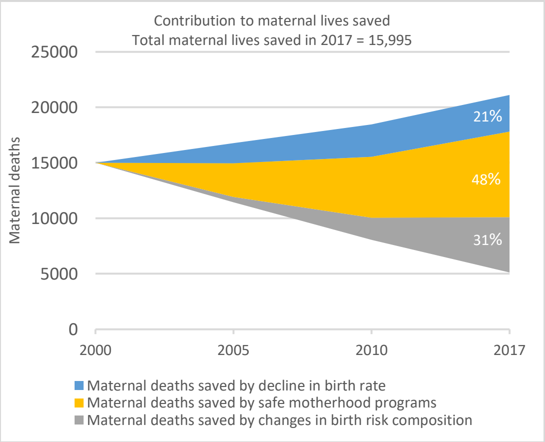

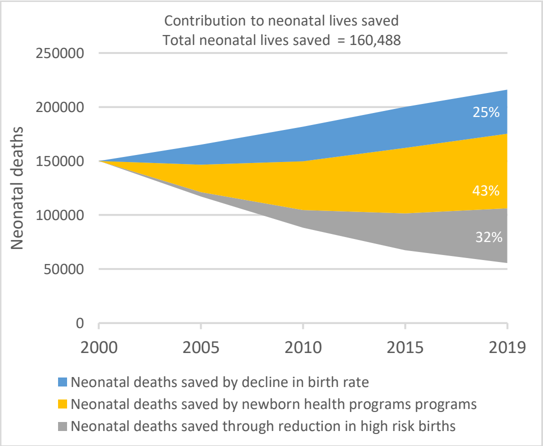

Note on interpretation: Assuming no change since the year 2000, the total number of maternal deaths or neonatal deaths will increase overtime given total births are also increasing. The top of the wedge is the total number of deaths if no change since 2000 and the bottom is the observed (actual) number of deaths. The different colors show the contribution of the three factors to the decline in deaths (decline in birth rate, safe motherhood programs, and changes in risk composition).

Annex Figure 14. Bangladesh Maternal Mortality Ratio 2000 to 2019, Lives Saved Tool (LiST) and United Nations Maternal Mortality Estimation Inter-agency Group (UN-MMEIG) estimate.

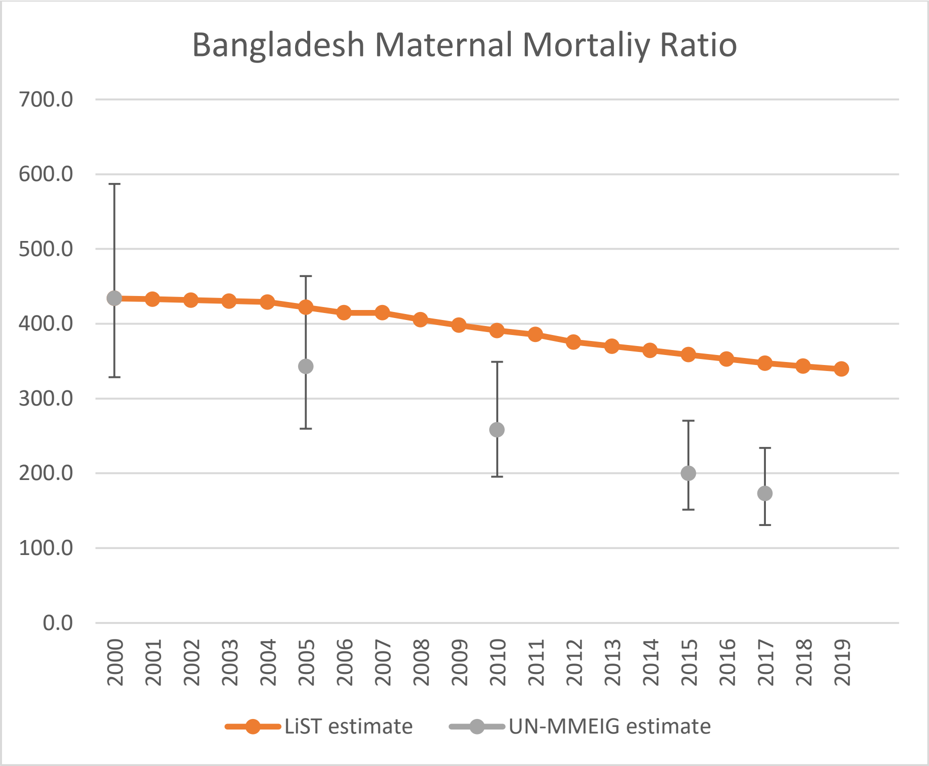

Annex Figure 15. Bangladesh Neonatal Mortality Rate 2000 to 2019, Lives Saved Tool (LiST) and United Nations Inter-agency Group for Child Mortality Estimation (IGME) estimate

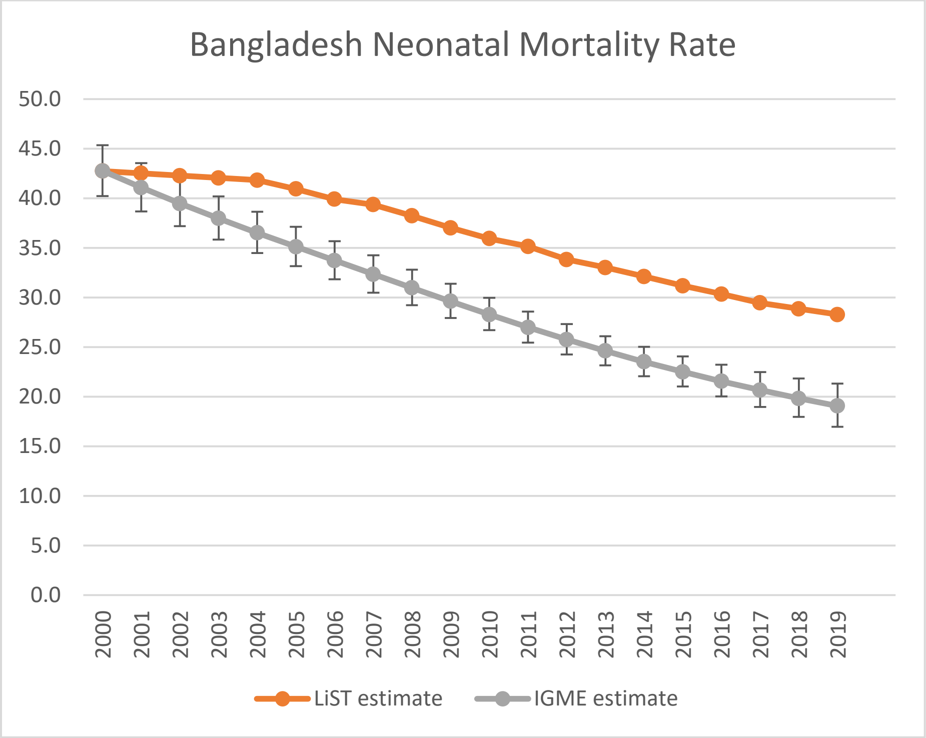

Annex Figure 16. Contribution of distal, intermediate and proximal factors to reduction of neonatal mortality (NMR) in Bangladesh

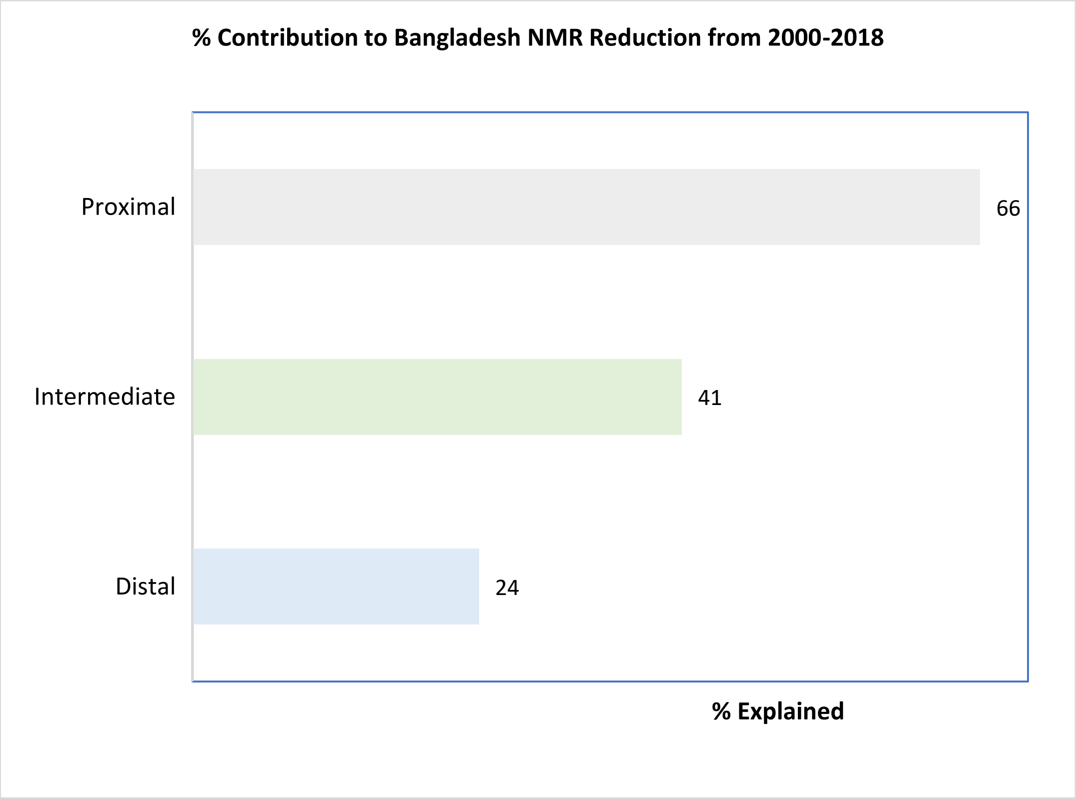

References

1. Aizawa T. Decomposition of Improvements in Infant Mortality in Asian Developing Countries Over Three Decades. Demography. 2021;58(1):137-63.
2. Bado AR, Appunni SS. Decomposing Wealth-Based Inequalities in Under-Five Mortality in West Africa. Iran J Public Health. 2015;44(7):920-30.
3. Demombynes G, Trommlerová SK. What has driven the decline of infant mortality in Kenya in the 2000s? Econ Hum Biol. 2016;21:17-32.
4. Dwomoh D, Amuasi S, Agyabeng K, Incoom G, Alhassan Y, Yawson AE. Understanding the determinants of infant and under-five mortality rates: a multivariate decomposition analysis of Demographic and Health Surveys in Ghana, 2003, 2008 and 2014. BMJ Glob Health. 2019;4(4):e001658.
5. Emamgholipour Sefiddashti S, Nakhae M, Kazemi Karyani A, Ghazanfari S. Decomposition Socioeconomic Inequality in Infant Mortality in EMRO Countries. International Journal of Pediatrics. 2015;3(4.1):749-56.
6. Goli S, Doshi R, Perianayagam A. Pathways of economic inequalities in maternal and child health in urban India: a decomposition analysis. PloS One. 2013;8(3):e58573.
7. Hong R, Ayad M, Rutstein SO, Ren R. Childhood mortality in Rwanda: Levels, trends, and differentials: Further analysis of the Rwanda Demographic and Health Surveys, 1992-2007/8. Calverton, Maryland, USA: ICF Macro; 2009.
8. Van Malderen C, Amouzou A, Barros AJ, Masquelier B, Van Oyen H, Speybroeck N. Socioeconomic factors contributing to under-five mortality in sub-Saharan Africa: a decomposition analysis. BMC Pub Health. 2019;19(1):1-19.
9. Hong R, Hor D. Factors associated with the decline of under-five mortality in Cambodia, 2000-2010: Further analysis of the Cambodia Demographic and Health Surveys. Calverton, Maryland, USA: ICF International; 2013.
10. National Institute of Population Research and Training (NIPORT) and ICF. Bangladesh Demographic and Health Survey 2017-18: Key Indicators. Dhaka, Bangladesh and Rockville, Maryland USA; 2019.
11. Singh NS, Blanchard AK, Blencowe H, Koon AD, Boerma T, Sharma S, et al. Zooming in and out: a holistic framework for research on maternal, late foetal and newborn survival and health. Health Policy and Planning. 2021.
12. United Nations Inter-Agency Group for Child Mortality Estimation (UN IGME). Levels and Trends in Child Mortality. Report 2020. Estimates developed by the UN Inter-agency Group for Child Mortality Estimation.
