# Supplementary material for: Effective multi-sectoral approach for rapid reduction in maternal and neonatal mortality: the exceptional case of Bangladesh
Source: BMJ Glob Health. 2024 May 6;9(Suppl 2):e011407. doi: 10.1136/bmjgh-2022-011407 (PMC11085986; doi:10.1136/bmjgh-2022-011407)
Supplement: Uncited online supplemental file 1 [file bmjgh-2022-011407supp002.pdf]

Authorship reflexivity statement

Engagement	1. Has the research team engaged constructively with the reflexivity statement?<br>We appreciate the opportunity to document this partnership with between two institutions, one based in Bangladesh and one based in the United States. One of the first authors, Elizabeth A Hazel responded to this statement on behalf of the other authors.
Co-development	2. Have the research partners co-developed the research study?<br>Yes, both institutions were involved in the development of the protocol, tools, data collection, analysis and manuscript writing.<br>3. Does the study address priority research questions for the LMIC partner(s)?<br>Yes, stakeholders in Bangladesh (public health researchers, practitioners, governmental officials and clinicians) are eager to understand the drivers of reduction in maternal and newborn mortality and how they can be sustained. This work also builds on previous exemplars analysis on child health in Bangladesh led by ICDDR,B.
Authorship	4. Is there a LMIC partner who is the first or last author?<br>Yes.<br>5. How have LMIC early career researchers been incorporated as authors?<br>Early career researchers from Bangladesh were instrumental and critical to the success of this research and the authorship for this manuscript accurately reflects their contributions.
Dissemination	6. How are data shared with LMIC partners to address research needs?<br>All data included in this manuscript have been shared between the two institutions.<br>7. Is there open access funding to improve publication dissemination?<br>Yes, we are pleased to have this manuscript published open access.
